# Functional Genomics of the Retina to Elucidate its Construction and Deconstruction

**DOI:** 10.3390/ijms20194922

**Published:** 2019-10-04

**Authors:** Frédéric Blond, Thierry Léveillard

**Affiliations:** Department of Genetics, Sorbonne Université, INSERM, CNRS, Institut de la Vision, 17 rue Moreau, F-75012 Paris, France; frederic.blond@inserm.fr

**Keywords:** retinal development, inherited retinal degenerations, retinitis pigmentosa, age-related macular degeneration, photoreceptors, comparative genomics, transcriptomics, proteomics, redox-biology

## Abstract

The retina is the light sensitive part of the eye and nervous tissue that have been used extensively to characterize the function of the central nervous system. The retina has a central position both in fundamental biology and in the physiopathology of neurodegenerative diseases. We address the contribution of functional genomics to the understanding of retinal biology by reviewing key events in their historical perspective as an introduction to major findings that were obtained through the study of the retina using genomics, transcriptomics and proteomics. We illustrate our purpose by showing that most of the genes of interest for retinal development and those involved in inherited retinal degenerations have a restricted expression to the retina and most particularly to photoreceptors cells. We show that the exponential growth of data generated by functional genomics is a future challenge not only in terms of storage but also in terms of accessibility to the scientific community of retinal biologists in the future. Finally, we emphasize on novel perspectives that emerge from the development of redox-proteomics, the new frontier in retinal biology.

## 1. Introduction

The vertebrate retina is composed of three layers of neurons, two layers of synapses and Müller glial cells which stretch radially across the thickness of the retina [1]. The outer nuclear layer contains the cell bodies of photoreceptor cells (rods and cones), the inner nuclear layer contains the cell bodies of the bipolar, horizontal and amacrine cells and the ganglion cell layer contains the cell bodies of the retinal ganglion cells and displaced amacrine cells. In contrast, the invertebrate retina is composed of regular hexagonal arrays, the ommatidia, each containing eight types of photoreceptors R1 to R8 [2,3]. Invertebrate eyes have microvilli-based rhabdomeric photoreceptors while vertebrate have cilia-based outer segment photoreceptors. These two fundamental types of photoreceptors have evolved unique structures to expand their apical membrane, an adaptation that help them to better accommodate their phototransduction machinery [4].

The eye and its photosensitive component, the retina, have fascinated evolutionary biologists for more than a century. The eye was perceived as a challenge to the theory of evolution due to its extreme perfection and complexity. Charles Darwin wrote that it is inconceivable to our imagination that the formation of a structure as perfect and complex as the eye could result from natural selection even if this may have been really achieved through numerous inherited gradations useful to animals [5]. Some authors proposed that the eyes of vertebrates and invertebrates arose independently, but the discovery that the transcription factor PAX6 serves as a master control gene for eye morphogenesis in insects and mammals finally demonstrated that the various eye-types are built with the same genetic tools, indicating their common phylogenetic origin [6,7].

The evolution of vision can be traced back to its putative origins in cyanobacteria a prokaryotic eubacteria found in the oldest known fossils on Earth, three and a half billions years ago. Cyanobacteria can sense the light–dark cycle and maintain oscillatory behaviors even under constant environmental conditions. This timing mechanism, called the circadian clock, involves in eukaryotes biochemical oscillators and multiple intertwined transcription/translation feedback loops. Cyanobacteria respond to light by phototactic motility behavior, a movement of a whole organism toward the light, or inversely avoidance of light [8]. Interestingly, in cyanobacteria, light perception does not rely on opsins but on redox-sensitive proteins of the thioredoxin-like superfamily, indicating that the circadian clock senses the cellular redox state rather than light intensity [9]. Peroxiredoxins, a class of highly conserved redox proteins that undergo 24-h redox cycles in both the unicellular green alga and in the enucleated human red blood cells, suggest that light perception was originally dependent upon that type of posttranslational oxidoreduction mechanisms [10,11]. In the animal kingdom, the discharge by cnidocytes, specialized photosensitive cells that the cnidarian *Hydra vulgaris* use for capturing prey, is regulated by light and opsin-mediated phototransduction [12]. Other cnidarians have sophisticated eyes with photoreceptor cells that transmit the light for perception to secondary neurons, representing thus the first stages of brain evolution. Therefore, we can assume that the eye as a sensory organ has evolved with the brain, providing sensory information subsequently processed by the brain. Consciousness has been suggested to have arisen from the evolution of light perception into vision [13]. It is not surprising that the evolution and the development of photoreceptors are a matter of interest for many biologists [14]. The present review illustrates the power of functional genomics toward our understanding of the retina illustrated by examples from studies on photoreceptor cells.

## 2. Retinal Development is Controlled by a Complex Gene Network

The power of drosophila genetics combined with microscopic examination of the phenotype of the drosophila eye has formed the basis of very powerful genetic screens. A block in photoreceptor cell differentiation triggers the roughening of the external eye surface [15]. *Son of sevenless* (SOS) was identified based of the ability of SOS alleles to suppress the eye phenotype of the *sevenless* mutant that carries an inactive receptor tyrosine kinase gene. Sevenless is essential for R7 photoreceptor differentiation [16]. SOS was further shown to be the missing link between two major classes of proto-oncogenes, the receptor tyrosine kinases and the ras family of GDP/GTP-binding proteins [17].

An integrated model of retinal cell-fate in the vertebrate retina was obtained using in vivo lineage tracing using retroviral vectors and autoradiographic tracers [18,19,20]. Numerous transcription factors of the basic helix-loop-helix (bHLH) family contribute to retinal cells fate and differentiation. Cone rod homeobox (CRX), an OTX-like homeobox gene was identified independently by two groups by degenerate RT-PCR or yeast one-hybrid screening as a gene important for photoreceptor differentiation [21,22]. The paired-type homeodomain transcription factor OTX2, a key regulator of the photoreceptor lineage, provides a necessary, but not sufficient signal to induce the photoreceptor cell fate. Early expression of CRX in postmitotic photoreceptor precursors is regulated by OTX2 [23]. Subtractive cDNA cloning led to the identification of neural retina leucine zipper protein (NRL), another class of transcription factors involved in photoreceptor differentiation [24]. NRL is required for rod photoreceptor development and regulates the expression of the orphan nuclear receptor NR2E3 [25,26]. The differentiation of cone photoreceptors relies on the action of the thyroid hormone receptor, a nuclear receptor regulated by binding to its ligand the thyroid hormone [27]. Thyroid hormone from extra ocular tissues is required for producing medium-wave cones and represses short-wave cone fate. Human infants with low thyroid hormone have an increased incidence of color vision defects. The developmental program of the retina depends on cell-autonomous and non-cell autonomous cues, but recent works on induced pluripotent cells demonstrates that the genetic program is robust enough to generate retinal organoids in vitro [28]. Interestingly, retinoblastoma, a juvenile eye tumor, originates from cone precursors [29,30,31]. The retinoblastoma susceptibility gene (*RB1*) was the first tumor suppressor gene identified [32,33,34]. The RB1 protein limits the transcription of cell cycle genes, primarily via regulation of the E2F transcription factor [35].

**Figure 1:**
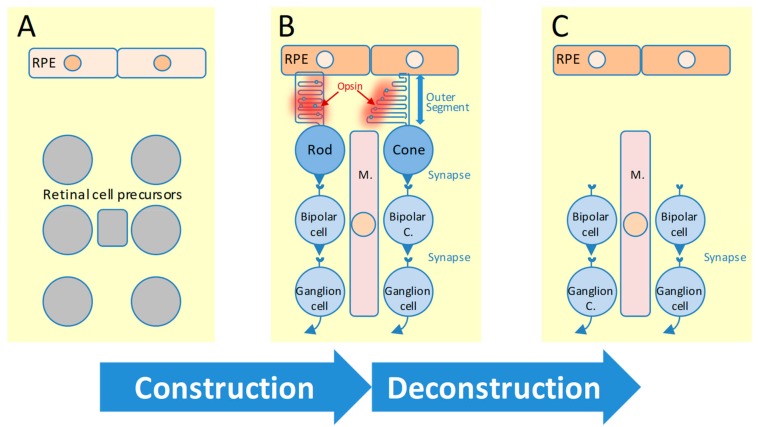
Construction and deconstruction of the retina. (**A**) The differentiation of retinal cell precursors into mature retinal cells relies on a complex gene network. (**B**) The function of the retina requires the transmission of information from photons captured by the outer segment of photoreceptors (rods and cones) to ganglion cells through, a process mediated by synaptic transmission via bipolar cells. The outer segment is renewed after its elimination by phagocytosis by the retinal pigmented epithelium (RPE). (**C**) Inherited retinal degenerations triggers the loss of rods and cones.

During the construction of the retina (Figure 1A,B), transcription factors are apparently not the only architects since programmed cell death is required to fine-tune the numerical balance between photoreceptors and other retinal cell types as shown by the role of ciliary neurotrophic factor (CNTF) [36]. The access of transcription factors to their recognition elements onto DNA is in addition regulated by epigenetic mechanisms [37,38]. The epigenetic control of gene regulation during retinal development is mediated by DNA methylation and posttranslational modifications of histones [39,40]. Methylation of the fifth carbon position of the cytosine residue in a 5’-CpG-3’ dinucleotide (CpG) confers a repressed state to the chromatin associated with the inhibition of gene expression [41]. Histones compact the DNA into nucleosomes and interfere with the accessibility of transcriptions factors to their recognition elements [42,43]. Assembly/disassembly of nucleosomes relies on the activity of two antagonistic class of enzymes, the histone acetylases and deacetylases [44]. Acetylation removes the positive charge on the histones, thereby decreasing the interaction of the N-termini of histones with the negatively charged phosphate groups of DNA. Histone acetylation denudes the DNA and consequently makes DNA-responsive elements more accessible to transcription factors. Histone deacetylase 4 and 1 are essential for rod differentiation [45,46]. Histone deacetylases of class III use NAD^+^ as a co-factor to deacetylate acetyl lysine residues of protein substrates, among which histones. Consequently, epigenetic control is tributary to the metabolism of the cell since NAD^+^ is produced among other sources by the reduction of pyruvate to lactate by lactate dehydrogenase A [47]. This is not a unique example of the intervention of cell metabolism in retinal development since endogenous lipid peroxidation produces secondary messengers that regulates retinal cell differentiation by redox signaling in zebrafish [48].

## 3. Inherited Retinal Diseases Caused by Photoreceptor Degeneration

A certificate of authenticity of the genes regulating retinal developmental is indirectly provided by their implication in inherited retinal dystrophies [49,50,51]. Inherited retinal degenerations are a group of genetic diseases in which a variety of mutations lead to vision loss and often blindness. The disease causing mutations often occur in genes that are critical for retinal function, leading to photoreceptor cell death (Figure 1B,C) and associated vision loss [52,53]. The number of new loci found to cause inherited retinal degenerations is increasing at a rate that matches the identification of causing genes, so that the actual total number of loci is 307, with 271 identified causative genes (https://sph.uth.edu/retnet/). Among the most common forms of such diseases, retinitis pigmentosa affects nearly 2 million people worldwide [54,55]. In retinitis pigmentosa, there a progression from night blindness which originates from the death of rod photoreceptors by apoptosis to the dysfunction of cone photoreceptors concentrated at the center of the retina, the fovea. This is a secondary event that can lead to complete blindness [56]. On the other hand, congenital stationary night blindness does not lead to the loss of central vision since rod bipolar cells are non-functional, but viable [57]. Cone rod dystrophies are characterized by primary cone involvement or by concomitant loss of both types of photoreceptors [58]. Stargardt disease causes progressive degeneration of the macula at the center of the retina [59]. Leber congenital amaurosis which affects simultaneously cones and rods is the most severe form of inherited retinal dystrophies [60,61]. Patients suffering of achromatopsia have little or no cone function and total absence of color perception [62,63,64]. These diseases constitute the major non-syndromic inherited retinal diseases [65]. Within the syndromic forms, Usher syndrome, a prevalent cause of inherited deafness also causes retinitis pigmentosa [66,67]. Bardet-Biedl syndrome is a ciliopathy characterized by retinal dystrophy along with obesity, polydactyly, renal failure, hypogonadism and cognitive impairment [68,69]. The genes that cause the disease participate in the formation of a stable protein complex named the BBSome involved in vesicular trafficking to the photoreceptor cilium [70].

The genetics of inherited retinal degeneration is very complex as illustrated here by the case of retinitis pigmentosa (RP) (Table 1). We examined the expression data of the genes known to cause RP and used literature searches for refinement. Expression data include the kinetics of the transcriptome of retina of the retinal degeneration-1 (*rd1*) mouse [71] as well as the transcriptome of human retinal detachment [72], the mouse tissue expression profiling publicly available and sequences from normalized libraries [73]. Among the retinitis pigmentosa genes whose profile could be examined, the rod-like expression pattern accounts for 45%. It should be noticed that because of the high proportion of rods (97%) over cones (3%) in the mouse retina, the expression profiles do not distinguish genes expressed specifically by rods (e.g., *RHO*) from those expressed by rods and cones (e.g., *RPGR*). Forty-eight percent of the genes examined from surgical specimens of human retinal detachment with both rod and cone death (PR-death), displays a similar rod-like expression pattern, confirming what observed in the *rd1* retina. We next considered that some of the rare genes are actually not carrying disease-causing mutations. We excluded genes with rare alleles, leaving us with 45 genes (50%) with a report of frequency of mutations in retinitis pigmentosa. Those 45 genes account for 53% of autosomal dominant, 45% autosomal recessive and 66% X-linked RP. Twenty-nine out of 40 genes whose profile could be examined, rod-like expression patterns account for 73%. Altogether and as expected, the listed genes carrying mutations causing retinitis pigmentosa is biased toward genes expressed by photoreceptors (rods and cones). Virtually all genes involved in the phototransduction cascade and in the vitamin A cycle carry mutations in individual patients suffering of inherited retinal degenerations [74,75]. Considering Leber congenital amaurosis, the only form of inherited retinal degeneration treated by corrective gene therapy [76,77,78,79,80,81], the *RPE65* gene was identified as a gene encoding for a protein of 65 kDa expressed specifically by the retinal pigmented epithelium [82]. Thereby, both biologists interested in retinal development and geneticists working on inherited retinal degenerations are focusing their attention on genes specifically expressed by retinal cells, with a special emphasis on photoreceptors.

## 4. Genomics and the Data Explosion

Many of the retinal genes of interest described above were identified in the pre-genomic times, but all the scientific community of biologists were eager to get the DNA script of the human and mouse genomes to facilitate their understanding of any biological scenarios under their scrutiny. Sequencing the entire human genome was a tedious task and its achievement has been compared to man’s first steps on the moon fifty years ago. Once the technological barrier had been overcome, the sequencing of other genomes became greatly facilitated [218,219]. Comparative genomics, the first field of research emerging from the sequencing of genomes has validated the unity of life and has provided robust arguments in favor of Darwin’s theory of evolution by natural selection [220]. Massive DNA sequencing has also spilled over to other disciplines, such as ecology and microbiology, known as ecogenomics and metagenomics. These sciences rely on the identification of individual species through their genome sequence in complex specimens of zooplankton or gut microbiome [221,222]. Genome sequencing is making an essential contribution to anthropology deciphering the complex admixture of ancestral *Homo* species and their migrations in prehistoric times [223,224,225,226,227]. Even ancient history related into the Bible has been studied by genomics showing the Philistines genome carries the signature of a population from southern Europe [228]. Comparative genomics addresses also the evolutionary origin of vision and of the central nervous system in the first animals, a debate that is far from closed [229,230,231,232,233]. Ctenophora (comb-jelly) and Porifera (sponge) are today the two candidates to the position of closest phylum to the first animal [234,235].

The exponential increase in the amount of data generated by genome sequencing has given birth to bioinformatics, later on to systems biology and nowadays to the concept of big data. The size of the genome of the entire human population is 2.42 × 10^19^ base pairs (bp), to which is added 4.15 × 10^24^ bp of the human microbiome. Biological sciences have an asymptotically reaching problem similar to that of the most powerful particle accelerator, the large hadron collider (LHC) of the European Council for Nuclear Research (CERN). LHC manages to store the data using the grid built on the technology of the World Wide Web (invented at CERN in 1989). Collisions in the LHC produce too much data to record, so they are filtered to retain only the interesting ones for analysis. Will this filtering be ethic for genomic data? This is an eminently complex question that will not be resolved here.

The retina is not in rest in this revolution. Age related macular degeneration (AMD) affects a region of the retina, the macula that is not present in non-primate species, and consequently a rodent model of AMD is still missing. This led researchers to concentrate their efforts on the genetic predisposition of AMD. AMD is a polygenic and polyfactorial disease as opposed to Mendelian inherited retinal degenerations, such as retinitis pigmentosa. Family and twin studies have demonstrated that the susceptibility for AMD is under the influence of the genome. Age and a positive family history of AMD are the two strongest risk factors for AMD. It has been shown that an individual with a sibling or a parent with AMD is 12–27 times more susceptible than someone from the general population to develop AMD. Geneticists have successfully employed the genetic variations in the sequence of the human genome among individuals to map loci that may contain genes carrying risk alleles for polygenic diseases [236]. Single nucleotide polymorphisms (SNPs), spread all over the genome, are used as genetic markers of causative alleles since alleles at a given locus are in linkage disequilibrium [237]. Genetics association studies search for difference in the frequency of each allele of SNP between a population of patients and another population of presumably healthy subjects. A difference signs the presence of a causative allele in a gene located in that locus. Genome wide association studies (GWAS) have led to the identification of several AMD susceptibility genes [238]. Variants in the complement factor H (*CFH*) gene on chromosome 1q32 have been associated with an increased risk for AMD [239,240,241]. These findings imply that the innate immune system may play a significant role in AMD pathogenesis. Several additional complement genes have also been associated with AMD reinforcing the role of the innate immune system in AMD pathology. One should notice that the analysis is performed without the possibility to follow the segregation of the risk alleles in a pedigree as done for Mendelian diseases. This is reflected in the difficulties that geneticists encounter in searches for the causal variants. Initial reports on *CFH* gene focused on the Y402H coding variant that alters a single amino acid in the CFH protein, but additional SNPs shows higher risk [242]. For the second major locus contributing to AMD, *ARMS2/HTRA1*, it has not yet been possible to determine if the AMD susceptibility results from the variants in the *ARMS2* or the nearby *HTRA1* gene or both [243,244]. A more complete picture of AMD was obtained with more statistical power by increasing the number of genotyped individuals by the Age-related Macular Degeneration Genomics Consortium (IAMDGC), coordinated by the NEI (http://amdgenetics.org/). IAMDGC performed a meta-analysis of the results of 14 GWAS representing > 17,100 advanced AMD cases and >60,000 controls of European and Asian ancestry. The meta-analysis examined 2,859,744 imputed and genotyped SNPs [245]. This massive approach has paid off and resulted in clear evidence for association in 19 regions of the genome each with at least one SNP with p < 5 × 10^−8^. Among these loci, 12 were previously associated with AMD, whereas seven were newly identified by the consortium [246]. The knowledge that has been gained was substantial. In an effort to identify AMD causative alleles, IAMDG consortium genotyped 16,144 patients and 17,832 controls for rare coding variations (exonic content) and variants relevant to AMD. Fifty-two independently associated common and rare variants, among which the lactate transporter gene *SCL16A8*, distributed across 34 loci were identified [247,248]. Overall, these variants are estimated to account for 40-60% of the genetic contribution to disease risk. Today, AMD susceptibility genes and environmental predictors (smoking, nutritional,…) are refined to target high risk individuals for heightened awareness, more frequent surveillance and clinical examinations, as well as identification of high-risk individuals for inclusion in clinical trials of novel therapies [249]. The therapeutic perspectives of this avenue of genetic research and the molecular mechanisms by which identified risk alleles cause increase disease risks are areas of intense study by retinal biologists.

## 5. Retinal Specific Expression Patterns Revealed by Transcriptomics

As illustrated above by examples in the field of research on the retina, both developmental biologists and human geneticists have a strong interest in genes with retina- and photoreceptor-restricted expression profiles. The selection is generally achieved by comparing global gene expression between specimens that represent two extreme conditions. This was originally done by subtractive and differential cloning using the degenerated *rd1* retina [250]. The concept evolved thereafter to cDNA arrays and to serial analysis of gene expression (SAGE) [251,252]. Rapidly the need for standardization became clear, resulting in part in the increased use of commercial oligo-arrays [72,112,253,254,255]. Each experiment generates data that are beyond what could be reported in a publication, so the research community created databases to score and exchange the increasing body of transcriptomic data. Examples include the Gene Expression Omnibus [256] ArrayExpress [257] and the ENCyclopedia Of DNA Elements (ENCODE) databases. The major shortfall in the application of ENCODE data for ocular research is in the relative paucity of eye tissue used for analysis [258]. The US Food and Drug Administration examined the expression array technology and concluded that the measurements were highly reproducible within and across the platforms, allowing the development, among others, of new prognostic and predictive tests for breast cancer [259,260]. The existence of numerous rodent models of inherited retinal degeneration led researchers to conduct comparisons between the transcriptomes of several models (Table 1). This includes spontaneous models, as the *rd1* mouse [261], retinal degeneration induced by light damage [262] or hypoxia [263], models obtained by inactivation of the retinal genes by homologous recombination [112,264] or by random integration of a dominant mutation in the rhodopsin gene [111,265], or even using conditional inactivation in a specific retinal cell type [266]. A model of eye morphogenesis was constructed using gene editing by Clustered, Regularly Interspaced, Short Palindromic Repeats-associated Endonuclease 9 (CRISPR/Cas9) [267]. The output of these comparisons is a list of common pathways underlying retinal degenerations and another list of genes pointed out by their specific expression by a subset of retinal cells [268,269]. Retinobase was developed as a web-based interface to provide efficient access to the global expression profiling of retinal genes from different organisms under various conditions [270,271]. The data can be visualized into radars that are very quickly interpreted because of our instinctive ability to recognize objects even when their form slightly change [272].

Soon after, retinal biologists got interested in gene expression in terms of alternative splicing because this phenomenon alters dramatically the function of the encoded proteins [273]. The motivation came also from the existence of mutations at the origin of inherited retinal degeneration in four genes encoding splicing factors [274] (Table 1). Attempts were made to accommodate microarray in the form of exon array, but the scientific community shifted rapidly to the technology of RNA sequencing (RNAseq) [275,276,277]. An advantage of RNAseq over microarray is that it counts the number of reads of each RNA species, while the microarray one scores the intensity of the signal captured after hybridization to a probe [278]. RNAseq also identifies unknown RNA species, like long noncoding RNAs [279]. Noncoding RNAs were known for a long time. Small nuclear ribonucleoproteins (snRNPs) are RNA-protein complexes that form the spliceosome. Small nucleolar RNAs (snoRNAs) are a class of small RNA molecules that primarily guide chemical modifications of other RNAs, as the ribosomal RNAs. Long non-coding RNAs (lncRNAs) participate in the regulation of gene expression at the transcription level and though epigenetic mechanisms. Both self-splicing RNAs (ribozymes) and microRNAs (miRNA) have the ability to silence gene expression [280,281,282,283]. The latter led retinal biologists to use them in a way to reduce the expression of dominant mutant rhodopsin proteins to establish a treatment for retinitis pigmentosa [284]. The sequence of artificial ribozymes is designed to target specifically the causative mutation among many others existing in the same gene (e.g., *RHO*). This restriction led other researchers to develop a broader approach using engineered miRNA, or small interfering RNA (siRNA) to suppress the expression of any dominant mutations in the rhodopsin gene by RNA interference and to replace the normal copy of that gene by its reintroduction using the same vector [285].

Biologists working on retinal development are constantly looking for cell specific markers that can be used to specify retinal cell fate during development to reveal its transcriptional code [269]. Various methods have been used to isolate total RNA from single cells of the retina including fluorescence-activated cell sorting [254,286]. The incorporation of tags to individual cells and massive parallel sequencing that is now used to avoid the need of single cell isolation [287,288,289,290,291]. This led to a comprehensive characterization of the gene regulatory networks that participate in initiation of neurogenesis, in developmental competence, and specification and differentiation of each major retinal cell type. This novel technology that captures single cell trajectory during retinal differentiation is very promising.

## 6. The Function of Retinal Proteins Assessed Using Proteomics

The assembly of genomes and their annotation allow the in silico translation of putative messenger RNA into proteins and deduce their molecular weight. By digesting a complex mixture of proteins by an endoproteinase, as trypsin, the individual mass of each small peptide of this collection can be accurately measured by mass spectrometry. By comparing the masses of these tryptic peptides with the in silico-digested proteins from the genome you can identify proteins in any protein mixture, even of proteins that have not yet been characterized [292,293]. Biologists working on the retina soon got interested in proteomics since the action of the genes involves their transcription into mRNAs that are then translated into proteins. The first successful attempts used the separation of individual proteins using two-dimensional (2D) gel electrophoresis [294]. This novel field of research is quite dependent on the construction of repository database and on bioinformatics tools such as those provided by the PRoteomics IDEntifications (PRIDE) database [295,296]. The proteome of the human eye is recognized as a project on its own [297].

Beside this repertoire of proteins expressed in the eye, differential proteomic analysis was quite successfully applied to the physiopathology of inherited retinal, degenerations by comparing disease specimens to healthy ones [298,299]. The approach was more rarely applied to retinal development most likely because the morphogens as the transcription factors, act at a low concentration and are difficult to detect by mass spectrometry [300,301,302]. One can further argue that transcriptomics is best suited to analyze developmental processes where the organ is constructed, but proteomics is a better option to study degenerative processes, during organ deconstruction. Indeed, while the developmental program starts by the transcription of a genetic program encoded by the genome, degeneration is the result of a genetic deficit that is initially perceived by the proteome. In the first studies, the tryptic peptides were mobilized after getting charged using matrix-assisted laser desorption/ionization and identified by the time-of-flight (MALDI-TOF) of the corresponding ions in a mass spectrometer. The only theoretical limitation comes from the fact that two amino-acids leucine and isoleucine cannot be distinguished because they have an identical mass. Nevertheless, tandem mass spectroscopy (MS/MS) is a real technological improvement since the sequence of each tryptic peptide can be identified after its fragmentation by collision [303,304]. This novel protocol became rapidly robust enough to replace the separation of proteins by 2D gels by a semi-purification by liquid chromatography (LC-MS/MS) [305]. The analysis was applied to clinical specimens of retinoblastoma [306,307] and this approach permitted the identification of many clinical biomarkers [308,309,310,311]. The approach was applied to retinal cell culture [312], but more significantly to subcellular compartments as the photoreceptor cilium and its outer segment [313,314]. Methods were also developed to analyze the distribution of proteins over the entire thickness of the retina [315,316].

Initially, proteomics was mainly semi-quantitative, the results were pointing to proteins that were more abundant in one of two groups of specimens by differential analysis, but the need for quantitative data was pressing. Diverse protocols were developed with the use of non-radioactive isotopes that allows the discrimination of tryptic peptides of identical sequences by the difference in their mass. One such approach relies on the incorporation of these isotopes in cell culture as the stable isotope labeling by amino acids in cell culture (SILAC) [317,318]. Another approach is based on the covalent labeling of the N-terminus and side chain amines of tryptic peptides with tags of varying isotopic masses after treating the specimens to get quantitative values, as for isobaric tags for absolute and relative quantification (iTRAQ) [319]. A real absolute quantification can be obtained by the use of an internal isotope-labeled peptide in a method called multiple reaction monitoring (MRM) [320].

Proteomics, which is a technology that does not use an amplification step as polymerase chain reaction (PCR), captures the most abundant protein of the cells, among which are the enzymes of the cellular metabolism. Metabolomics is an approach that measures the concentration of metabolites in a given specimen and was also applied to retinal specimens to decipher their metabolic status [321,322,323,324,325]. Interestingly, certain metabolites can be visualized in situ by mass spectrometric imaging [326,327]. Metabolomics belongs to functional genomics in a sense that it provides deep investigation of the content of metabolites in a specimen but it does not emerge from genomics since most of the metabolites were identified in the pre-genomic time by the use of biochemistry and radioactive tracers [328]. One exception comes from the chemical diversity of plants with their ~200,000 secondary metabolites, by far more numerous than for animal species. This goes beyond the scope of this review but it is interesting to notice than in that specific case, the sequence of the genome can be used as a valid information [329].

It is certainly thanks to the fact that mass spectrometry is capable of identifying unambiguously most of the protein content of a protein fraction of interest that proteomics became widely used by retinal biologists to identify protein-protein interactions. The diverse methods used to purify these fractions rely mostly on the question addressed. The use of an antibodies against the bait protein is an appropriate choice for co-immunoprecipitation [71,330,331,332,333] and immunoaffinity purification [334,335,336]. Alternatively, the bait protein is immobilized and the proteins interacting with it are co-purified by affinity chromatography [337]. In other circumstances, the interacting proteins are identified from a list of candidates that share the same mobility after gel electrophoresis. This is the case of protein overlay assay and far-western blotting [71,338]. Proteomics has advantageously replaced yeast two-hybrid screening as a method of choice when looking for protein interactors [339,340]. The approach is becoming so robust than proteomics is now used to identify proteins that are located in the vicinity of a protein bait using proximity-dependent labeling methods for proteomic profiling [341]. These interactions define protein regulatory modules that participate in cell signaling.

Since it is clear to all biologists that cell signaling is regulated at the post-translational level, researchers got interested by post-translational modifications [342]. The complete list of physiologically relevant modifications of amino acids of proteins is quite large so we will concentrate here only a limited number of examples. We have mentioned above histone acetylation/deacetylation that is amenable to proteomic studies [343]. Phosphorylation is taking a central place in cell signaling with hundreds of kinases and phosphatases that act in cascade to control cellular activity by the post-translational modifications of three amino acids: serine, threonine and tyrosine of retinal proteins [344,345,346] and retinoblastoma [347]. Methods have been developed to enrich phosphopeptides and to facilitate their characterization such as immobilized metal affinity chromatography (IMAC) [348]. Negatively charged phosphate groups of peptides from phosphoproteins interact with positively charged metal ions (Fe^3+^, Ga^3+^, and Al^3+^) and this interaction makes it possible to enrich phosphorylated peptides from rather complex peptide samples. Phosphoproteomics is still a method that is more time consuming than the use of antibodies raised against phosphopeptides, but it is much more precise.

Another type of post-translational modifications is generated by reactive oxygen species (ROS). Due to the probable involvement of ROS in neurodegenerations, which include the inherited retinal degenerations and aging related diseases, such as AMD, a plethora of studies relating damages of oxidation on macromolecules in the pathology of the retina have been published. Retinal proteins can be modified irreversibly by 4-hydroxynonenal, a product of lipid peroxidation [349]. Carboxyethyl pyrrole, a unique oxidation fragment of docosahexaenoic acid forms adducts with retinal proteins in AMD more than in the healthy retina [350]. Another type of oxidations became the focus of retinal biologists since they are reversible [351,352,353]. Cysteines and methionines can be reversibly oxidized, and their redox status is involved in cell signaling. In recent years, the role of cysteine residues as redox sensors in cell signaling pathways has gained increasing attention. Proteomic studies aimed at large-scale identification of proteins with modified cysteines have provided tools for unraveling new redox-regulated processes, a domain named disulfide proteomics or redox proteomics [354]. The reactive nature of cysteine thiols is often an experimental challenge when determining the in vivo cysteine oxidation state of proteins. In biological samples, postlysis thiol-disulfide exchange may lead to misinterpretations of data. For example, if molecular oxygen is present in the buffers, oxidation of C-SH may occur during isolation. Hence, one of the critical steps in redox biology is to trap the thiol-disulfide status. This is achieved by a direct alkylation producing a first fraction of stable thiol derivatives from the reduced thiols within the protein extract. The oxidized thiols are not reactive to this alkylation. Post-alkylation the oxidized thiols are reduced, and then alkylated producing a second fraction of stable thiol derivatives. The challenge is to distinguish the two fractions. Initially, this was achieved by using different fluorescence probes visualized after separation by 2-D difference gel electrophoresis (Redox DIGE) [355]. But, as stated above, gel electrophoresis has many potential problems and methods relying on differential mass tags have imposed themselves, as isotope coded affinity tag (ICAT) [356,357,358]. To “freeze” the thiol state of the proteins before alkylation, proteins are precipitated using trichloroacetic acid. The widely used alkylating reagents for blocking free C-SH are 2-iodoacetamide (IAM) or N-ethylmaleimide (NEM) and the resulting tryptic peptides identified and quantified in a relative manner by mass spectrometry. Two distinct isotope of the alkylating agent can also be used [359]. The method has not yet been successfully applied to the retina but efforts to develop robust protocols are ongoing.

## 7. Conclusions

Retinal biologists are now immerged in the post-genomics area. Questions concerning the development of the retina and its physiopathology are becoming more and more complex and the results of each investigation generally contain data that is far more complete than what was the focus of each study. It is of great interest to the community of biologists to keep track of these data that could be analyzed posteriorly by experts in their respective field. This objective is not trivial due to the exponential growth of the volume of data generated by a single functional genomics experiment. Biology is being confronted by the same trend than is also challenging the society: the increase in the amount of information is not proportional to our understanding of it. Nevertheless, functional genomics is a great leap forward to the future.

## Figures and Tables

**Table 1:** The expression of genes mutated in retinitis pigmentosa.

N	Inheritance	Gene	*Rd1* [71]	RD [72]	Mouse Tissue	RPE/PR [73]	Freq.	Other Ret. Dis.	Other OMIM	Mouse Models	Other Models.
1	**Autosomal dominant**	*ARL3*	Rod-like	PR-death	Neurons	NF	Rare [83]	No	No	[84]	No
2		*ADIPOR1*	Rod-like	PR-death	Bone mar.	Ret./RPE	Rare [85]	One	No	[86]	No
3		*BEST1*	Hom	Infl.	Testis	NF	Rare [87]	Four	No	[88]	Dog [89,90]
4		*CA4*	NE	NOp	Intestine	NF	Rare [91]	No	No	No	No
5		*CRX*	Rod-like	PR-death	Ret./RPE	Ret./RPE	1% [54]	Four	No	[92]	Cat [93]
6		*FSCN2*	Rod-like	NOp	Ret./RPE	Retina	3% [94]	One	No	[95]	No
7		*GUCA1B*	Rod-like	PR-death	Ret./RPE	Ret./RPE	0-5% [94]	One	No	[96]	No
8		*HK1*	Rod-like	Hom	Testis	Retina	Rare [97]	No	142600	No	No
9		*IMPDH1*	Rod-like	PR-death	Ret./RPE	Retina	2–3% [94]	One	No	[98]	No
10		*IMPG1*	Rod-like	PR-death	Ret./RPE	Retina	Rare [99]	Two	No	No	No
11		*KLHL7*	Hom	Hom	Ub	Ret./RPE	1–2% [100]	No	611119	No	No
12		*NR2E3*	Rod-like	PR-death	Ret./RPE	Retina	3.5% [101]	Four	No	[102]	No
13		*NRL*	Rod-like	PR-death	Ret./RPE	Retina	2% [94]	One	No	[25]	No
14		*PRPF3*	Hom	PR-death	Ub	Ret./RPE	1% [94]	No	No	[103]	No
15		*PRPF4*	Hom	Hom	Ub	RPE	Rare [104]	No	No	No	ZebF [105]
16		*PRPF6*	Hom	Hom	Ub	Ret./RPE	Rare [104]	No	No	No	No
17		*PRPF8*	Hom	PR-death	Ub	Ret./RPE	2% [94]	No	No	[103]	No
18		*PRPF31*	Rod-like	Hom	Ub	NF	2–4% [94]	No	No	[106]	No
19		*PRPH2*	Rod-like	PR-death	Ret./RPE	Ret./RPE	1–8% [94]	Six	No	[107,108]	No
20		*RDH12*	Rod-like	PR-death	Ret./RPE	Ret./RPE	Rare [109]	One	No	[110]	No
21		*RHO*	Rod-like	PR-death	Ret./RPE	Ret./RPE	2–26% [94]	Two	No	[111,112,113]	[114,115]
22		*ROM1*	Rod-like	PR-death	Ret./RPE	Ret./RPE	1% [94]	One	No	[116]	No
23		*RP1*	Rod-like	PR-death	Ret./RPE	RPE	4–8% [94]	No	No	[117,118]	No
24		*RP9*	Cone-L.	PR-death	Ub	Retina	Rare [94]	No	No	No	No
25		*RPE65*	Hom	NE	RPE	NF	Rare	Two	No	[104]	Dog [76]
26		*SEMA4A*	Cone-L.	NOp	Ub	Ret./RPE	Rare [119]	One	No	[120]	No
27		*SNRNP200*	Hom	Hom	Ub	NF	<2% [121]	No	No	No	No
28		*SPP2*	NE	NOp	Kidn. Liv.	NF	Rare [122]	No	No	No	No
29		*TOPORS*	Hom	Hom	Ub	RPE	Rare [123]	No	No	[124]	No
30	**Autosomal recessive**	*ABCA4*	Rod-like	PR-death	Ret./RPE	Ret./RPE	5–6% [54]	Four	No	[125]	Dog [126]
31		*ADIPOR1*	Rod-like	PR-death	Bone Mar.	Ret./RPE	Rare	One	No	[86]	No
32		*AGBL5*	Hom	Hom	Testis	Retina	Rare [127]	No	No	No	No
33		*AHR*	Rod-like	Hom	Mast cells / Ret.	NF	Rare [128]	No	No	[129]	No
34		*ARHGEF18*	Hom	Hom	White cells / RPE	Ret./RPE	Rare [130]	No	No	No	No
35		*ARL6*	Rod-like	PR-death	Ub	Retina	1% [54]	One	No	[131]	No
36		*ARL2BP*	Hom	Hom	Testis	Ret./RPE	Rare [132]	No	No	No	No
37		*BBS1*	Rod-like	Hom	Ret./RPE	RPE	2-3% [54]	One	No	[133]	No
38		*BBS2*	Rod-like	Hom	Ub	Ret./RPE	0.8% [54]	One	No	[134]	No
39		*BEST1*	Hom	Infl.	Testis	NF	Rare [87]	Four	No	[88]	Dog [89,90]
40		*C2orf71*	NF	PR-death	NF	NF	1% [135]	No	No	[136]	No
41		*C8orf37*	No orth.	NOp	No ortho	No ortho	Rare [137]	Three	No	No	No
42		*CERKL*	Rod-like	NF	Ub	NF	1% [54]	One	No	[138]	No
43		*CLCC1*	Hom	Hom	Ub	Retina	Rare [139]	No	No	[139]	No
44		*CLRN1*	NOp	Infl.	NOp	NF	1% [54]	One	No	[140]	ZebF [141]
45		*CNGA1*	Rod-like	PR-death	Ret./RPE	Retina	1% [54]	No	No	No	Xen [142]
46		*CNGB1*	NF	PR-death	NF	Ret./RPE	4% [54]	No	No	[143]	Dog [144]
47		*CRB1*	Rod-like	Hom	Ret./RPE	NF	1% [54]	Three	No	[145]	Rat [146]
48		*CYP4V2*	Cone-L.	Hom	Liver	NF	Rare [147]	One	No	[148]	No
49		*DHDDS*	Hom	PR-death	Ub	Ret./RPE	1–8% [149]	No	No	No	ZebF [150]
50		*DHX38*	Hom	Hom	Ub	Retina	Rare [151]	One	No	No	No
51		*EMC1*	NF	Hom	NF	NF	Rare [152]	No	616846	No	No
52		*EYS*	No orth.	NOp	No ortho	No ortho	[153,154]	No	No	No ortho	ZebF [155]
53		*FAM161A*	Rod-like	PR-death	Ret./RPE	NF	2% [156]	No	No	[157]	Dog [158]
54		*GPR125*	Hom	Hom	Epidermis	NF	Rare [152]	No	No	No	No
55		*HGSNAT*	Cone-L.	Hom	Microglia	Retina	Rare [159]	No	610453	No	No
56		*IDH3B*	Hom	Hom	Adipo.	Ret./RPE	Rare [160]	No	No	No	No
57		*IFT140*	Hom	Hom	Bone mar.	NF	Rare [161]	Two	614620	[162]	No
58		*IFT172*	Hom	PR-death	Testis	NF	Rare [163]	One	607386	[164]	No
59		*IMPG2*	NF	Hom	NF	RPE	Rare [165]	One	No	No	No
60		*KIAA1549*	No orth.	PR-death	No ortho	NF	Rare [152]	No	No	No	No
61		*KIZ*	Rod-like	NF	Testis	NF	Rare [166]	No	No	No	No
62		*LRAT*	Infl.	NOp	RPE	RPE	1% [54,167]	One	No	[168]	No
63		*MAK*	Rod-like	PR-death	Ret./RPE	RPE	[149,169]	No	No	[170]	No
64		*MERTK*	Cone-L.	Hom	Ub	RPE	1% [54]	No	No	[171]	[172]
65		*MVK*	Hom	Hom	Ub	Retina	Rare [173]	No	251170	[174]	No
66		*NEK2*	Cone-L.	NOp	Ub	NF	Rare [175]	No	No	[176]	ZebF [175]
67		*NEUROD1*	Rod-like	Hom	Cerebel.	NF	Rare [177]	No	601724	[178]	No
68		*NRL*	Rod-like	PR-death	Ret./RPE	Retina	2% [94]	One	No	[25]	No
69		*PDE6A*	Rod-like	PR-death	Ret./RPE	Retina	3–4% [54]	No	No	[179]	Dog [180]
70		*PDE6B*	Rod-like	PR-death	Ret./RPE	RPE	4–5% [54]	One	No	*rd1, rd10*	Dog [181]
71		*PDE6G*	Rod-like	PR-death	Ret./RPE	NF	Rare [182]	No	No	[183]	No
72		*POMGNT1*	Hom	Hom	Saliv. gl.	RPE	Rare [184]	No	606822	[185]	No
73		*PRCD*	No orth.	PR-death	No ortho	No ortho	Rare [186]	No	No	No ortho	Dog [187]
74		*PROM1*	Rod-like	PR-death	Ub	Ret./RPE	Rare [188]	Four	No	[186]	No
75		*RBP3*	Rod-like	PR-death	Ret./RPE	Ret./RPE	Rare [189]	No	No	[190]	No
76		*REEP6*	Rod-like	PR-death	Liver/Ret./RPE/Testis	RPE	Rare [191]	No	No	[192]	No
77		*RGR*	Infl.	PR-death	RPE	RPE	0.5% [54]	One	No	[193]	No
78		*RHO*	Rod-like	PR-death	Ret./RPE	Ret./RPE	1% [54]	Two	No	*Rho-/-*	No
79		*RLBP1*	Cone-L.	PR-death	Ret./RPE	Ret./RPE	1% [54]	Three	No	[194]	No
80		*RP1L1*	Rod-like	PR-death	Ret./RPE	NF	0.5% [195]	One	No	[196]	No
81		*RPE65*	Hom	NE	RPE	NF	2% [54]	Two	No	[104]	Dog [76]
82		*SAG*	Rod-like	PR-death	Ret./RPE	Ret./RPE	>1% [54]	One	No	[197]	Dog [198]
83		*SAMD11*	Rod-like	Hom	Bone mar. Ret./RPE	RPE	Rare [199]	No	No	No	No
84		*SLC7A14*	Cone-L.	PR-death	Brain	NF	Rare [200]	No	No	[200]	No
85		*SPATA7*	Hom	Hom	Testis	RPE	Rare [201]	One	No	[202]	No
86		*TRNT1*	Hom	NOp	Ub	NF	Rare [203]	No	612907	No	ZebF [204]
87		*TTC8*	Rod-like	PR-death	Ub	NF	>1% [54]	One	No	[205]	Dog [206]
88		*TULP1*	Rod-like	PR-death	Ret./RPE	Retina	1% [54]	One	No	[207]	No
89		*USH2A*	Rod-like	PR-death	Ub	NF	17% [54]	One	No	[208]	No
90		*ZNF408*	No orth.	Infl.	No ortho	No ortho	Rare [209]	One	No	No	ZebF [210]
91		*ZNF513*	No orth.	Hom	No ortho	No ortho	Rare [211]	No	No	No	ZebF [211]
92	**X-linked**	*OFD1*	Hom	PR-death	Ub	RPE	Rare [212]	Two	300170	[213]	ZebF [214]
93		*RP2*	Hom	NOp	Ub	NF	7–10% [12]	No	No	[215]	No
94		*RPGR*	NF	PR-death	NF	NF	80% [12]	Three	No	[216]	Dog [217]

Adipo.: Adipocytes, Bone mar.: Bone Marrow, Cerebel.: Cerebellum, Cone-L.: Cone-like: increase in the *rd1* retina (after rod degeneration) and decrease in human retinal detachment (after death of both rods an cones), Freq: Frequency from Retnet (https://sph.uth.edu/Retnet/) and Pubmed, Hom: Homogenous, Infl.: Inflammatory cells, Kidn.: Kidney, Liv.: Liver, NE: Not expressed, NF: Not found, NOp: Probeset non-operational, No ortho: No orthologue, PR-death: Decrease in human retinal detachment, Ret.: retina, Rod-like: decrease in the *rd1* retina. Rod-like expression pattern corresponds to an expression profile that march that of the rhodopsin gene (*Rho*) that decrease tin the rd1 retina during the course of rod degeneration., RPE: retinal pigmented epithelium, Saliv. gl.: Salivary gland, Ub: Ubiquitous, Xen: Xenopus and ZebF: Zebrafish.

## Abbreviations

AMD	Age-related macular degeneration
ARMS2	Age-Related Maculopathy Susceptibility 2
bHLH	Basic helix-loop-helix
CERN	Conseil européen pour la recherche nucléaire - European Organization for Nuclear Research
CFH	Complement factor H
CNTF	Ciliary neurotrophic factor
CpG	Cytosine-phosphate-guanine
CRX	Cone rod homeobox
GDP/GTP	Guanosine di/tri phosphate
GWAS	Genome-wide association study
HTRA1	HtrA Serine Peptidase 1
LHC	Large hadron collider
NAD+	Nicotinamide adenine dinucleotide
NRE2E3	Nuclear receptor subfamily 2, group E, member 3
NRL	Neural retina leucine zipper
OTX	Orthodenticle homeobox
PAX6	Paired Box 6
RB1	Retinoblastoma 1
*rd1*	Retinal degeneration-1
RHO	Rhodopsin
ROS	Reactive oxygen species
RP	Retinitis pigmentosa
RPE	Retinal pigmented epithelium
RPE65	Retinal Pigment Epithelium-Specific 65 KDa Protein
RPGR	Retinitis pigmentosa GTPase regulator
SLC16A8	Solute Carrier Family 16 Member 8
SOS	Son of sevenless
